# Prevalence and Risk Factors of Self-Reported Symptoms Consistent with Carpal Tunnel Syndrome Among Dentists in Jordan

**DOI:** 10.3390/jcm14186630

**Published:** 2025-09-20

**Authors:** Loiy Khasawneh, Ahmad Aldardour, Mohammad Olimat, Shefa’a Alnammneh, Salah Tewfik Daradkeh, Mohammad Nammaneh, Wesam A. Debes, Ahmad R. Al-Qudimat

**Affiliations:** 1 Department of Special Surgery, Faculty of Medicine, The Hashemite University, Zarqa 13133, Jordan; 2 Physical Therapy Department, Rumailah Hospital, Hamad Medical Corporation, Doha 3050, Qatar; 3 Orthopedic Surgery Department, Ministry of Health, Amman 11118, Jordan; olimat.mohammad@live.com; 4 Independent Researcher, Irbid 22110, Jordan; shefaanaamneh@gmail.com; 5 Special Surgery Department, Faculty of Medicine, Yarmouk University, Irbid 21163, Jordan; salah.daradkeh@yu.edu.jo; 6 Private Dental Clinic, Irbid 21410, Jordan; dr.naamneh91@yahoo.com; 7 Physiotherapy Department, Faculty of Allied Medical Sciences, Applied Science Private University, Amman 11931, Jordan; wesamcm2019@gmail.com; 8 Surgical Research Section, Department of Surgery, Hamad Medical Corporation, Doha 3050, Qatar; aalqudimat@hamad.qa; 9 Department of Public Health, College of Health Sciences, QU-Health, Qatar University, Doha 2713, Qatar

**Keywords:** carpal tunnel syndrome, dentists, occupational health, musculoskeletal disorders, Boston Carpal Tunnel Questionnaire

## Abstract

**Background**: Dentists are at an increased risk of developing musculoskeletal disorders, particularly carpal tunnel syndrome (CTS), due to repetitive hand movements, awkward postures, and sustained grip forces. This study aimed to investigate the prevalence and risk factors of self-reported wrist and hand symptoms and clinically relevant CTS indicators among dentists in Jordan. **Methods:** A cross-sectional study was conducted among 201 licensed dentists in Jordan. Participants completed demographic questionnaires and the Valid Arabic version Boston Carpal Tunnel Questionnaire (BCTQ) to assess their symptom severity and hand function. Data was analyzed using STATA version 17, applying descriptive statistics, chi-squared tests, t-tests, ANOVA, and multivariable linear regression to evaluate association between sociodemographic factors and BCTQ scores. **Results:** Of the 201 participants, 64.2% were female and 35.8% were male. Female dentists were significantly younger (median age 31 vs. 39 years, *p* < 0.001), reported higher symptom severity (median score 18.0 vs. 16.0, *p* = 0.019), and experienced greater functional limitations (median score 15 vs. 9, *p* < 0.001) than male dentists. The overall mean symptom severity score was 19.12 (SD = 7.82), and the functional impairment score was 14.20 (SD = 6.37), indicating mild pain and functional limitation. Multivariable regression revealed that male sex was associated with significantly lower symptom scores (β = 0.7, *p* = 0.001) and better function (β = 0.722, *p* = 0.002). Geographic location, higher education level (PhD), and full-time employment were associated with higher symptom scores in the study. **Conclusions:** Wrist and hand symptoms are prevalent among dentists in Jordan, with significant sex differences in symptom severity and functional impairment. Geographic location, academic degree, and working hours were significant predictors of CTS-related symptoms. These findings underscore the need for ergonomic interventions and targeted preventive strategies, especially for high-risk groups, such as female and full-time practitioners.

## 1. Introduction

Carpal tunnel syndrome (CTS), resulting from compression of the median nerve within the carpal tunnel, is the most prevalent peripheral nerve entrapment disorder, affecting millions of individuals worldwide [1,2,3,4]. It is characterized by symptoms such as paresthesia, dysesthesia, sensory loss, and weakness and atrophy of the thenar muscles in advanced stages. Although symptoms are typically confined to the hand, they may extend proximally to the forearm, upper arm, and even the shoulder [5]. The prevalence of CTS is estimated to be approximately 5% in the general population [1,4,6,7]. Risk factors for CTS include repetitive and excessive use of the wrist and hand, awkward postures, heavy lifting, and exposure to vibrations, as well as personal factors such as female sex, obesity, older age, and smoking [3,4,8,9,10,11,12]. Furthermore, the increasing incidence of systemic metabolic disorders, including diabetes, thyroid dysfunction, and obesity, has exacerbated the burden on healthcare systems worldwide, as these conditions are recognized as contributors to CTS, particularly in stressful occupational environments [13,14,15]. The prevalence of work-related CTS varies across occupations, with higher rates observed in jobs necessitating repetitive or forceful hand use [2,4,6,16]. Dentistry is considered a high-risk occupation because of the repetitive wrist movements involved in clinical procedures such as root canal treatment, scaling, tooth preparation, and extraction. Previous studies have demonstrated that dentists are more likely to develop CTS than the general population [17,18,19,20,21,22]. However, data on the prevalence of CTS among dentists in developing countries, including Jordan, are limited. According to the Jordanian Dental Association, there were approximately 10,500 practicing dentists in Jordan in 2023, underscoring the importance of investigating this issue in this expanding professional group. This study aimed to assess the prevalence of CTS among dentists in Jordan and identify the factors associated with this syndrome. We hypothesized that CTS symptoms are more prevalent among dentists than in the general population. The significance of this study lies in exploring the severity of CTS symptoms among dentists, which may assist orthopedic and hand surgeons in identifying preventive measures and reducing the severity of CTS. Ultimately, this could decrease the need for surgical interventions, electrodiagnostic testing, and prolonged sick leave in these patients. To our knowledge, no study has previously assessed the prevalence and risk factors of self-reported wrist and hand symptoms and clinically confirmed CTS among dentists in Jordan.

## 2. Method

A cross-sectional study was conducted in Jordan in 2024, focusing on male and female workers who had been employed for a minimum of one year. Dentists with a history of orthopedic trauma or congenital deformities were excluded from the study. Additionally, the sample size was determined using a 95% confidence level and a 5% margin of error, resulting in an estimated requirement of approximately 383 participants. However, this target proved impractical during the recruitment phase because of time and resource constraints. Consequently, to establish a more feasible and transparent estimate, we recalculated the sample size using the same confidence level but with a 7% margin of error, which necessitated approximately 192 participants in the study. The final enrollment of 201 participants satisfied the recalculated requirement.

The questionnaire surveys were administered either through in-person interviews conducted by research team members with dentists present during the data collection period or distributed electronically via email as a Google Form or through social media platforms. Ethical approval was obtained from the Institutional Review Board (IRB) of the Hashemite University.

Prior to engaging participants for the study (approval no. 23/3/2023/2024), comprehensive information regarding the study was conveyed to eligible patients before their enrollment. The questionnaire included an invitation letter detailing the study’s title, objectives, and contributions. Participation involved completing a brief online questionnaire, with assurances that responses would remain confidential and be used exclusively for academic purposes. Consent to participate was implied by completing the questionnaire. The Boston Carpal Tunnel Questionnaire (BCTQ), initially developed by Levine et al. [23], was employed to assess dentists regarding the symptoms and functional impacts of carpal tunnel syndrome (CTS). This instrument has been validated in prior research, exhibiting excellent test–retest reliability and intraclass correlation coefficients ranging from 0.8 to 0.9 [24,25,26]. The BCTQ is as effective as electrodiagnostic testing in predicting the presence of CTS [27]. The study questionnaire comprised three sections: demographic and health data, the Functional Status Scale (FSS), and the Symptom Severity Scale (SSS). Demographic and health data collected included age, sex, marital status, height, weight, smoking status, exercise habits, dominant hand, specialty, years of work, daily patient contact time, pre-existing illnesses or conditions, degrees other than dentistry, and previous diagnoses. The FSS evaluates the level of difficulty in performing eight functional tasks (writing, buttoning clothing, holding a book while reading, clutching a telephone handle, opening jars, home duties, carrying a supermarket basket, and bathing and dressing). The SSS comprised 11 questions addressing CTS symptoms throughout a typical day (severity of hand pain at night, pain in the hand during the day, duration of discomfort, hand numbness, weakness in the hand, numbness at night, and difficulties gripping small items). A rating scale of 1–5 was used, with 1 indicating no functional difficulty or symptoms and 5 indicating an inability to complete the functional task or maximal symptoms. The total score for each subscale was calculated by dividing the absolute score by the number of items: 11–55/11 for the SSS and 8–40/8 for the FSS.

## 3. Statistical Analysis

Statistical analyses were performed using STATA version 17. Descriptive statistics were used to summarize the participants’ characteristics. The normality of the distribution of continuous variables was evaluated using the Shapiro–Wilk test. Variables that were normally distributed, such as height, are reported as mean (SD), whereas variables that were not normally distributed, including age, weight, pain score, and function score, are presented as medians (IQR). Categorical variables, such as sex, marital status, education level, smoking status, job status, and governorate, were summarized using frequencies and percentages. Comparisons between groups were conducted using statistical tests appropriate for the distribution of the data: independent-samples *t*-tests for normally distributed continuous variables, Mann–Whitney U tests for non-normally distributed continuous variables, and chi-square tests for categorical variables. To assess differences across multiple governorates, one-way ANOVA was utilized when assumptions were satisfied, and Kruskal–Wallis tests were applied otherwise. Multivariable linear regression analyses were performed to investigate the relationship between sociodemographic factors and the two primary outcomes, pain score and function score, as assessed by the Boston Carpal Tunnel Questionnaire. The analyses reported the regression coefficients, standard errors, t-values, 95% confidence intervals (CIs), and corresponding *p* values. The geographic analysis encompassed 11 governorates, and Bonferroni correction was applied to adjust for multiple tests. The corrected significance threshold was set at *p* < 0.0045 (0.05/11). Unadjusted *p*-values are presented in the tables for transparency, and results that remained significant after Bonferroni correction are emphasized in Section 4. For all other analyses, statistical significance was set at *p* < 0.05.

## 4. Results

### 4.1. Demographic Characteristics of the Participants

**Figure 1:**
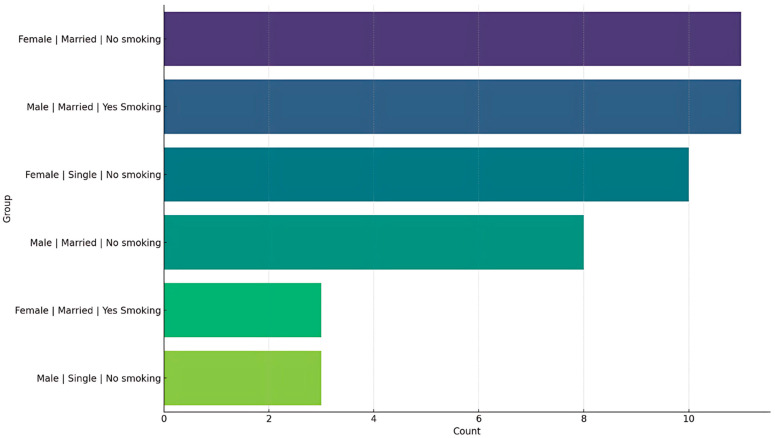
Distribution by gender, martial, and smoking status.

In a cohort of 201 participants, females represented a larger proportion (n = 129, 64.2%) than males (n = 72, 35.8%). The median age of females was 31 years (IQR: 28–35), whereas males were significantly older, with a median age of 39 years (IQR: 30.5–51). Males were, on average, significantly taller (mean = 176.6 cm, SD = 6.2) and heavier (median = 85.0 kg, IQR: 78.5–100.0) than females (mean height = 162.4 cm, SD = 5.8; median weight = 61.0 kg, IQR: 56.0–69.0 kg). The majority of the participants were married (57.7%), with a higher proportion of married males (69.4%) than females (51.2%). A notable sex disparity was evident in smoking status, with nearly half of the men (48.6%) identifying as smokers, in contrast to only 13.2% of women. Most participants resided in Amman (52.2%) and Irbid (30.3%), with minimal representation from other governorates. In terms of pain perception, females reported higher median pain scores (18.0, IQR: 13–25) than males (16.0, IQR: 11–21). Conversely, males exhibited lower median function scores (9 vs. 15), indicating superior hand function among males, as lower scores indicate less difficulty. Regarding educational attainment, a higher proportion of females possessed a bachelor’s degree (65.1%) compared to males (47.2%), while PhD qualifications were more prevalent among males (13.9% vs. 3.9%) (*p* = 0.008). Employment status also varied significantly by sex (*p* = 0.015), with 83.3% of males employed full-time compared to 67.4% of females (Table 1) (Figure 1).

### 4.2. Correlation Between Gender and Outcomes

Statistically significant sex differences were observed across several variables. Age, height, weight, pain score, and function score all demonstrated strong associations with sex (*p* < 0.001), indicating distinct demographic and clinical differences between male and female participants. Marital status also exhibited significant variation (*p* = 0.041), with a higher likelihood of marriage in men. Smoking status revealed the most pronounced disparity, with a significantly greater prevalence among men than among women (*p* < 0.001). Additionally, both education level (*p* = 0.008) and employment status (*p* = 0.015) were significantly associated with gender, with males more frequently holding advanced degrees and being employed full-time than females were. However, no significant differences were observed in the governorate of residence (*p* = 0.58), suggesting a balanced geographic distribution across sexes. Finally, although females reported higher pain scores, males exhibited superior hand function, as evidenced by lower functional difficulty scores (*p* < 0.001) (Table 1).

### 4.3. Boston Carpal Tunnel Syndrome Questionnaire (BCTQ) Score

#### 4.3.1. Pain Score Description

Participants reported a mean symptom severity score of 19.12 (SD = 7.82) out of a possible 55, equating to an average of approximately 1.74 per item on a 1–5 scale. According to the BCTQ symptom scale, this score indicates slightly mild pain symptoms. The most frequently reported symptoms included daytime pain frequency (mean = 2.01) and pain during the day (mean = 1.98), suggesting that discomfort was more prevalent during waking hours. Other symptoms, such as tingling sensations (mean = 1.76), numbness (mean = 1.73), and nocturnal pain (mean = 1.73), also contributed to the overall symptom burden but remained within the “slight” category. These findings suggest that while participants experienced hand and wrist symptoms, the overall pain severity was low to moderate, confirming that the study population, on average, reported the presence of pain symptoms, albeit not at a high-severity level.

#### 4.3.2. Function Score Description

The overall mean functional impairment score was 14.20 (SD = 6.37) out of a maximum of 40, corresponding to an average of 1.78 per item on the BCTQ function scale. This score is situated between the categories of “little difficulty” and “moderate difficulty,” suggesting that the majority of participants experienced only minor limitations in daily hand function. Tasks such as bathing and dressing (mean = 1.32), buttoning clothes (mean = 1.37), and writing (mean = 1.60) were assessed as the least challenging, whereas more physically demanding tasks, such as carrying a grocery basket, exhibited the highest difficulty (mean = 2.81), approaching the “moderate” range (Table 2).

#### 4.3.3. Multivariable Linear Regression Analysis

Regression analysis identified several significant predictors of pain scores among participants. Male participants exhibited significantly lower pain scores than female participants (β = 0.7, *p* = 0.001), indicating an association between the male sex and reduced pain severity. Among the governorates, residing in Al-Balqa (β = 1.425, *p* = 0.012), Al-Karak (β = 1.581, *p* = 0.001), and Zarqa (β = 0.746, *p* = 0.034) were significantly associated with pain scores. Specifically, individuals in Al-Balqa and Al-Karak reported significantly higher pain, whereas those in Zarqa reported significantly lower scores. Furthermore, participants from Tafila demonstrated significantly lower pain scores (β = 0.684, *p* = 0.001). After applying a Bonferroni correction for multiple testing across 11 governorates (adjusted significance threshold: *p* < 0.0045), only the associations with Al-Karak and Tafila remained statistically significant, while those with Al-Balqa and Zarqa did not meet the Bonferroni-adjusted threshold. In terms of educational attainment, individuals with a PhD reported significantly higher pain scores than those with a bachelor’s degree (β = 1.244, *p* = 0.017). Employment status was also influential, with part-time workers experiencing lower pain scores than full-time workers (β = 0.886, *p* = 0.049). Other variables, including age, height, marital status, and smoking, were not statistically significant. Weight was borderline significant (β = 1.003, *p* = 0.091), indicating a potential positive trend towards higher weight being associated with increased pain. (Table 3A). The regression model used to analyze the factors associated with hand function identified several significant associations. Male participants exhibited significantly lower function scores than female participants (β = 0.722, *p* = 0.002), indicating superior hand functionality among males, as lower scores denoted less difficulty on the Boston Carpal Tunnel Questionnaire (BCTQ) scale. Several governorates demonstrated significant differences in functional outcomes compared to the reference group. Specifically, residents of Jarash (β = 0.726, *p* = 0.027), Zarqa (β = 0.670, *p* < 0.001), Ajloun (β = 0.663, *p* = 0.012), and Mafraq (β = 0.782, *p* = 0.023) exhibited significantly better function (lower scores), whereas residents of Tafila had worse functional scores (β = 1.267, *p* = 0.025), suggesting increased difficulty in hand-related tasks. Other sociodemographic factors, including marital status, education level, smoking status, and job type, were not significantly associated with functional scores. Similarly, age, height, and weight were not significant predictors, although weight approached borderline significance (*p* = 0.108). (Table 3B).

## 5. Discussion

This study offers significant insights into the prevalence and characteristics of carpal tunnel syndrome (CTS) symptoms among dental practitioners in Jordan. Participants reported symptom severity scores on the Boston Carpal Tunnel Questionnaire (BCTQ), indicative of mild to moderate discomfort, with functional impairment also within the mild range. These findings suggest that while symptoms are not disabling, they are sufficiently present to impact routine activities of daily living. To the best of our knowledge, this is the first study in the region to utilize a validated instrument for the quantitative assessment of CTS in dentists.

### 5.1. Gender-Related Differences

Gender emerged as one of the most significant findings. Male dentists reported significantly lower pain scores (β = 0.7, *p* = 0.001) and better functional outcomes (β = 0.722, *p* = 0.002) than females, in contrast to general population studies, in which females typically exhibit a higher CTS risk. Possible explanations include age differences, anthropometric characteristics, and reporting bias.

### 5.2. Educational Level

Educational level also emerged as a factor, with dentists holding PhD degrees reporting higher pain scores (β = 1.244, *p* = 0.017). Part-time employment was associated with lower pain levels than full-time work (β = 0.886, *p* = 0.049), supporting a dose–response relationship between workload and CTS symptoms. Geographic variations were observed, with dentists in Al-Balqa and Al-Karak reporting higher pain scores, while those in Zarqa and Tafila reported lower scores. These differences may reflect variations in patient load, practice resources or ergonomic training availability. Symptom patterns indicated that daytime pain frequency and discomfort were the most common, consistent with occupational CTS. Functional impairments were most noticeable in tasks requiring sustained grip strength, such as carrying grocery baskets, whereas basic activities such as bathing and dressing were minimally affected.

### 5.3. Comparison with Literature

Our findings align with those of international studies demonstrating the substantial burden of CTS among dentists. Reported prevalence varies widely: 10.31% in Pakistan, 30.5% in Riyadh, 17% in Iran, 11% in Australia, and as high as 61.3% in Libya. Such variations may be attributed to differences in diagnostic tools, study designs, or occupational settings. The observed sex effect contrasts with general population studies, in which females consistently demonstrate a higher risk. This discrepancy may reflect occupation-specific dynamics or sociocultural factors influencing symptom reporting. The prevalence and severity observed in our study exceeded the estimates in the general population, which is approximately 5%, thereby reinforcing the occupational nature of CTS in dentistry.

### 5.4. Study Strengths and Limitations

Our study had several notable strengths, including the utilization of a validated instrument (BCTQ) with excellent reliability [23,24,25,26,27], diverse geographic representation across Jordan, and comprehensive demographic data collection. The sample size of 201 participants ensured adequate power for meaningful statistical analysis, with a margin of error of 7%.

However, several limitations of this study warrant acknowledgment. First, the cross-sectional design precludes causal inference and the ability to track symptom progression over time. Second, the use of a convenience sampling strategy introduced the potential for systematic selection bias. Dentists experiencing existing musculoskeletal or carpal tunnel symptoms may have been more inclined to participate, whereas those with heavier workloads or minimal symptoms may have been underrepresented. Additionally, the use of mixed recruitment channels (in-person, email, and social media) may have disproportionately attracted younger or digitally engaged dentists, thereby limiting the representativeness of the sample. These biases can systematically influence prevalence estimates and generalizability. Third, the assessment of symptoms relied on self-reported measures, which are susceptible to recall or reporting bias, and objective diagnostic confirmation (e.g., nerve conduction studies) was not conducted. Fourth, the exclusion of dental interns may have resulted in an underestimation of CTS prevalence, as younger practitioners in training could experience acute symptoms during skill development and adaptation to repetitive procedures. Finally, the study did not capture occupational details such as specialty, procedure type, workload intensity, or ergonomic training, which may serve as important risk modifiers. Despite these limitations, this study offers valuable insights into the symptoms of CTS among Jordanian dentists and underscores the necessity for larger, probability-based studies that incorporate objective diagnostics and detailed occupational exposures.

## 6. Practical Implications

The findings emphasize the critical need for preventive strategies to address carpal tunnel syndrome (CTS) in dental practice. Implementing ergonomic training, effective workload management, and regular occupational health screenings is essential to mitigate the symptom burden. The observed sex-related differences indicate that interventions may require customization for specific subgroups. The correlation between full-time employment and increased symptoms supports the adoption of policies that promote job rotation, reduced working hours, and mandatory rest periods. Geographic disparities underscore the necessity for targeted regional interventions to enhance occupational health awareness and ergonomic resources in areas with a higher burden.

## 7. Future Research Directions

Future research should employ longitudinal designs to track the development and progression of CTS in dental professionals. The integration of objective diagnostic measures with self-reported tools would enhance validity. Future studies should also assess the efficacy of ergonomic interventions and workplace modifications tailored to dental practice. The unexpected associations with sex and educational level warrant further exploration, as does the paradoxical finding that the prevalence of smoking among male dentists coincided with improved functional outcomes.

## 8. Conclusions

This study revealed a significant burden of CTS-related symptoms among dentists in Jordan, with notable sex differences and geographic variations. Although symptom severity was generally mild to moderate, the occupational nature of these symptoms and their impact on daily function underscore the necessity of comprehensive occupational health interventions. The findings advocate the implementation of preventive strategies, ergonomic enhancements, and targeted health screening programs for dental professionals to alleviate the burden of work-related musculoskeletal disorders in this high-risk occupation. The results contribute to the limited literature on CTS among dental professionals in developing countries and provide a foundation for evidence-based occupational health policies in the Jordanian dental profession. Ongoing surveillance and intervention research are crucial for addressing this significant occupational health challenge and safeguarding the long-term health and productivity of dental professionals in Jordan and similar contexts.

## Figures and Tables

**Table 1:** Participant characteristics. The star indicates statistical significance.

	Female N = 129	Male N = 72	Total N = 201	*p*-Value
**Age Median (IQR)**	31 (28–35)	39 (30.5–51)	32 (28–40)	<0.001 *
**Height Mean ± SD**	162.4 ± 5.8	176.6 ± 6.2	167.5 ± 9.0	<0.001 *
**Weight Median (IQR)**	61.0 (56.0–69.0)	85.0 (78.5–100.0)	68.0 (60.0–82.0)	<0.001 *
**Education level**				0.008 *
**Bachelor**	84 (65.12)	34 (47.22)	118 (58.71)	
**Master**	40 (31.01)	28 (38.89)	68 (33.83)	
**PhD**	5 (3.88)	10 (13.89)	15 (7.46)	
**Job status**				0.015 *
**Full-time**	87 (67.44)	60 (83.33)	147 (73.13)	
**Part-time**	42 (32.56)	12 (16.67)	54 (26.87)	
**Marital Status N(%)**				0.041 *
***Single***	59 (45.7%)	21 (29.2%)	80 (39.8%)	
***Married***	66 (51.2%)	50 (69.4%)	116 (57.7%)	
***Divorce***	4 (3.1%)	1 (1.4%)	5 (2.5%)	
**Smoking N(%)**				<0.001 *
***No***	112 (86.8%)	37 (51.4%)	149 (74.1%)	
***Yes***	17 (13.2%)	35 (48.6%)	52 (25.9%)	
**Governorate**				0.58
***Irbid***	39 (30.2%)	22 (30.6%)	61 (30.3%)	
***Al-Balqa***	3 (2.3%)	2 (2.8%)	5 (2.5%)	
***Amman***	73 (56.6%)	32 (44.4%)	105 (52.2%)	
***Jerash***	4 (3.1%)	4 (5.6%)	8 (4.0%)	
***Aqaba***	1 (0.8%)	2 (2.8%)	3 (1.5%)	
***Al-Karak***	3 (2.3%)	3 (4.2%)	6 (3.0%)	
***Zarqa***	1 (0.8%)	3 (4.2%)	4 (2.0%)	
***Ajloun***	2 (1.6%)	1 (1.4%)	3 (1.5%)	
***Madaba***	1 (0.8%)	2 (2.8%)	3 (1.5%)	
***Tafila***	1 (0.8%)	0 (0.0%)	1 (0.5%)	
***Mafraq***	1 (0.8%)	1 (1.4%)	2 (1.0%)	
**Pain score**	18.00 (13–25)	16.00 (11–21)	17.00 (13–24)	0.019 *
**Function score**	15	9	12	<0.001 *

**Table 2:** Distribution of participants in pain and function scores (N = 201).

Pain Questions		
Questions	Mean	SD
How severe is the hand or wrist pain that you have at night?	1.73	0.90
How often did hand or wrist pain wake you up during a typical night in the past two weeks?	1.37	0.69
Do you typically have pain in your hand or wrist during the daytime?	1.98	0.89
How often do you have hand or wrist pain during daytime?	2.01	0.85
How long on average does an episode of pain last during the daytime	1.92	0.86
Do you have numbness (loss of sensation) in your hand?	1.73	0.89
Do you have a weakness in your hand or wrist?	1.78	0.97
Do you have tingling sensations in your hand?	1.76	0.93
How severe is numbness (loss of sensation) or tingling at night?	1.76	0.94
How often did hand numbness or tingling wake you up during a typical night during the past two weeks?	1.51	0.83
Do you have difficulty with the grasping and use of small objects such as keys or pens?	1.53	0.85
Overall pain	19.12	7.82
**Function questions**		
Writing	1.60	1.17
Buttoning of clothes	1.37	0.94
Holding a book while reading	1.78	1.25
Gripping of a telephone handle	1.77	1.22
Opening of jars	1.81	1.20
Household chores	1.74	1.19
Carrying of grocery basket	2.81	1.24
Bathing and dressing	1.32	0.88
Overall function	14.20	6.37

SD: Standard Deviation.

**Table 3:** Multivariable regression variables with (**A**). Pain score, (**B**). Function score.

Variables	Coefficients	SE	t-Value	*p*-Value	95% Lower CI	95% Upper CI
**(A). Pain Score**						
**Age**	1.003	0.004	0.72	0.469	0.996	1.01
**Gender**						
***Female***	1	-	-	-	-	-
***Male***	0.7	0.068	−3.70	0.001 *	0.579	0.845
**Marital Status**						
***Single***	1	-	-	-	-	-
***Maride***	0.965	0.063	−0.55	0.586	0.848	1.097
***Divorce***	0.767	0.118	−1.73	0.084	0.567	1.036
**Smoking**						
***No***	1	-	-	-	-	-
***Yes***	0.986	0.063	−0.23	0.821	0.869	1.118
**Education level**						
***Bachelor***	1	-	-	-	-	-
***Master’s***	1.042	0.066	0.65	0.513	0.92	1.18
***PhD***	1.244	0.113	2.40	0.017	1.04	1.487
**Job status**						
***Full-time***	1		-	-	-	-
***Part time***	0.886	0.055	−1.96	0.049	0.785	1
**Height**	1.004	0.005	0.81	0.42	0.995	1.013
**Weight**	1.003	0.002	1.69	0.091	0.999	1.007
**Governorate**						
***Irbid***	1	-	-	-	-	-
***Al-Balqa***	1.425	0.201	2.52	0.012 *	1.081	1.877
***Amman***	1.011	0.063	0.18	0.855	0.895	1.143
***Jarash***	1.095	0.146	0.68	0.496	0.843	1.421
***Aqaba***	0.981	0.167	−0.11	0.911	0.703	1.37
***Al-Karak***	1.581	0.217	3.34	0.001 *	1.208	2.068
***Zarqa***	0.746	0.103	−2.12	0.034 *	0.568	0.979
***Ajloun***	0.965	0.099	−0.35	0.73	0.789	1.18
***Madaba***	1.087	0.166	0.54	0.588	0.805	1.467
***Tafila***	0.684	0.073	−3.55	0.001 *	0.554	0.844
***Mafraq***	1.008	0.14	0.05	0.956	0.767	1.324
**(B). Function Part**						
**Age**	1	0.004	0.02	0.985	0.992	1.008
**Gender**						
***Female***	1	-	-	-	-	-
***Male***	0.722	0.076	−3.08	0.002	0.587	0.888
**Marital Status**						
***Single***	1	-	-	-	-	-
***Maride***	1.035	0.076	0.46	0.642	0.896	1.194
***Divorce***	0.753	0.13	−1.64	0.1	0.537	1.056
**Smoking**						
***No***	1	-	-	-	-	-
***Yes***	1.07	0.083	0.87	0.384	0.918	1.247
**Education level**						
***Bachelor’s***	1	-	-	-	-	-
***Master’s***	1.034	0.069	0.51	0.61	0.908	1.178
***PhD***	1.036	0.134	0.28	0.782	0.805	1.334
**Job status**						
***Full-time***	1	-	-	-	-	-
***Part time***	0.933	0.067	−0.97	0.333	0.811	1.074
**Height**	0.996	0.005	−0.80	0.424	0.986	1.006
**Weight**	1.003	0.002	1.61	0.108	0.999	1.007
**Governorate**						
***Al-Balqa***	1.047	0.228	0.21	0.832	0.683	1.605
***Amman***	0.922	0.061	−1.21	0.226	0.809	1.051
***Jarash***	0.726	0.105	−2.21	0.027	0.547	0.964
***Aqaba***	0.931	0.192	−0.34	0.731	0.622	1.396
***Al-Karak***	1.196	0.227	0.95	0.344	0.825	1.735
***Zarqa***	0.67	0.063	−4.27	0	0.558	0.805
***Ajloun***	0.663	0.109	−2.50	0.012	0.48	0.914
***Madaba***	0.966	0.244	−0.14	0.892	0.589	1.584
***Tafila***	1.267	0.133	2.25	0.025	1.031	1.557
***Mafraq***	0.782	0.085	−2.27	0.023	0.632	0.967

Note: *p*-values are unadjusted. Statistical significance was assessed against a Bonferroni-corrected threshold (*p* < 0.0045 for 11 governorate comparisons). Associations remaining significant after correction are marked with *.

## Data Availability

The data presented in this study are available on request from the corresponding author due to privacy and ethical restriction and confidentiality.
